# Observations on the Use of Tobacco as a Local Application in Gout, and Other Cases of Constitutional Inflammation

**Published:** 1831

**Authors:** 


OBSERVATIONS ON THE USE OF TOBACCO AS A LOCAL APPLICATION IN GOUT, AND OTHER CASES OF CONSTITUTIONAL INFLAMMATION. By JOHN VETCH, M. D., Physician to the Charter-house.

[Med.-Chir. Trans. Vol. XVI.]

THIS paper is so very concise that we need not abridge it.

Under other circumstances it had been my intention to give to the public a series of detailed cases to establish the beneficial effects of tobacco as a local application, and one capable of alleviating in a great degree, and of sometimes altogether arresting various forms of specific inflammation, more particularly gout and rheumatic inflammation attacking synovial membrane. Besides the power which this vegetable possesses in allaying the pain and abating the inflammation of gout, it assists the parts most materially in recovering their tone and strength.

The sensible effects of tobacco upon the skin and cuticle are readily perceived, by immersing, for a short time, the fingers in an infusion, or in a watery solution of the extract.

The infusion forms a valuable application in all cases of erysipelatous inflammation, and the only precaution to be attended to, is not to apply it to any part contiguous to the stomach, unless the production of nausea be at the same time desirable.

I was led to appreciate the valuable sedative and astringent power of tobacco in the first instance, by the benefit I derived from it in cases of the last-mentioned class, having many years ago instituted an extensive trial of all the known narcotics, with the expectation of deriving additional aid in the treatment of purulent ophthalmia.

The good and the powerful effects which I obtained from the tobacco, fully compensated for the inefficiency of all the other local applications I then tried; its effects were notorious to all who saw it employed, and I now, as I ought to have done twenty years sooner, recommend its use to general notice, in cases of acute migratory inflammation, and especially when it attacks the joints, testicle, or sclerotic coat of the eye.

The infusion as directed by the London Pharmacopœia is sufficiently strong, and in many cases it is well to rub the part with eau de cologne after the use of the tobacco.

*Charter House, 28th Feb.* 1831.

---

XXII.

